# Simultaneous Determination and Pharmacokinetic Study of Losartan, Losartan Carboxylic Acid, Ramipril, Ramiprilat, and Hydrochlorothiazide in Rat Plasma by a Liquid Chromatography/Tandem Mass Spectrometry Method

**DOI:** 10.3797/scipharm.1410-15

**Published:** 2014-11-30

**Authors:** Ramkumar Dubey, Manik Ghosh

**Affiliations:** Department of Pharmaceutical Sciences and Technology, Birla Institute of Technology, Mesra, Ranchi, India

**Keywords:** LC-MS/MS, Simultaneous estimation, Validation, Plasma, Pharmacokinetic

## Abstract

The monitoring of the plasmatic concentrations of cardiovascular drugs is crucial for understanding their pharmacokinetics and pharmacodynamics. A simple, sensitive, specific, and high-throughput liquid chromatography/tandem mass spectrometry (LC–MS/MS) method was developed and validated for the simultaneous estimation and pharmacokinetic study of losartan (LOS), losartan carboxylic acid (LCA), ramipril (RAM), ramiprilate (RPT), and hydrochlorothiazide (HCZ) in rat plasma using irbesartan (IBS) and metolazone (MET) as internal standards (ISs). After solid phase extraction (SPE), analytes and ISs were separated on an Agilent Poroshell 120, EC-C18 (50 mm × 4.6 mm, i.d., 2.7 μm) column with a mobile phase consisting of methanol/water (85:15, v/v) containing 5 mmol/L ammonium formate and 0.1% formic acid at a flow rate of 0.4 mL/min. The precursor → product ion transitions for the analytes and ISs were monitored on a triple quadrupole mass spectrometer, operating in the multiple reaction monitoring (MRM) mode and switching the electrospray ionization (ESI) mode during chromatography from positive (to detect LOS, LCA, RAM, RPT, and IBS) to negative (to detect HCZ and MET). The method was validated as per the FDA guidelines and it exhibited sufficient specificity, accuracy, and precision. The method was found to be linear in the range of 3–3000 ng/mL for LOS and LCA, 0.1–200 ng/mL for RAM and RPT, and 1–1500 ng/mL for HCZ. The described method was successfully applied to the preclinical pharmacokinetic study of analytes after oral administration of a mixture of LOS (10 mg/kg), RAM (1 mg/kg), and HCZ (2.5 mg/kg) in rats.

## Introduction

To improve blood pressure control, the European hypertension guidelines recommend that angiotensin II receptor blockers (ARBs) or angiotensin-converting enzyme inhibitors (ACEIs) are combined with calcium channel blockers (CCBs) and/or thiazide diuretics [1]. The rationale for this strategy is based, in part, on their different effects on the renin-angiotensin system, which improves antihypertensive efficacy [2–5].

Losartan (LOS), chemically known as (2-butyl-4-chloro-1-{[2’-(1*H*-tetrazol-5-yl)[1,1’-biphenyl]-4-yl]methyl}-1*H*-imidazol-5-yl)methanol monopotassium salt, is an orally active non-peptide angiotensin II receptor antagonist [6]. It has a more potent active metabolite losartan carboxylic acid (LCA), chemically known as 2-butyl-4-chloro-1-{[2’-(1*H*-tetrazol-5-yl)[1,1’-biphenyl]-4-yl]methyl}-1*H*-imidazole-5-carboxylic acid [7–9].

Ramipril (RAM), (2*S*,3a*S*,6a*S*)-1-[(2*S*)-2-{[(2*S*)-1-ethoxy-1-oxo-4-phenylbutan-2-yl]amino}propanoyl]octahydrocyclopenta[*b*]pyrrole-2-carboxylic acid, is an orally active inhibitor of angiotensin converting enzyme (ACE), which is a prodrug used in the treatment of all forms of hypertension, heart failure, and following myocardial infarction to improve survival in patients with clinical evidence of heart failure [10, 11]. The active diacid metabolite, ramiprilat, (2*S*,3a*S*,7a*S*)-1-[(2*S*)-2-{[(2*S*)-1-ethoxy-1-oxopentan-2-yl]amino}propanoyl]octahydro-1*H*-indole-2-carboxylic acid, is formed by hydrolysis of its ethyl ester from ramipril [12].

Hydrochlorothiazide (HCZ) is a benzathiadiazine diuretic, chemically known as 6-chloro-1,2,3,4-tetrahydro-2,4-benzothiadiazine-7-sulfonamide 1,1-dioxide, used in hypertension and often prescribed in combination with other antihypertensive drugs such as beta blockers, angiotensin-converting enzyme inhibitors, or angiotensin II receptor blockers [13, 14].

Various analytical methods have been described for the determination of LOS and its metabolite LCA, alone or in combination with other drugs in a biological matrix including HPLC [15], LC-MS/MS [16–26]. Similarly, several analytical methods were reported in the literature for RAM and its metabolite RPT, alone or in combination with other drugs in a biological matrix, which include HPLC [27], LC-MS/MS [28–42], and UPLC-MS/MS [43]. Quantification of HCTZ in biological fluids was also reported using LC-MS/MS [45–49]. In terms of a combination of LOS, RAM, and HCZ, there is one method that has been reported by HPLC [50].

Due to the relatively low dosing range of LOS, RAM, and HCZ, it was necessary to develop a simple and sensitive method for the simultaneous quantification of these analytes and their metabolites in plasma at very low concentrations. Although, there have been many works reported on the quantification of these analytes alone or in combination with other drugs in biological fluids using diverse analytical techniques. To the best of our knowledge, there is no report on the use of LC–MS/MS for the simultaneous determination of LOS, LCA, RAM, RPT, and HCZ in rat plasma. The main objective of our research work was to develop a simple, sensitive, and high-throughput LC-MS/MS method for the simultaneous estimation of these analytes in rat plasma, which has a simple extraction procedure from small volumes of plasma, high recoveries, and a short run time. The method was successfully applied to the preclinical pharmacokinetic studies of these analytes after oral administration of a mixture of LOS (10 mg/kg), RAM (1 mg/kg), and HCZ (2.5 mg/kg) in rats.

## Experimental

### Chemicals and Reagents

LOS (purity > 99.60%), RAM (purity > 98.90%), RPT (purity > 99.10%), HCZ (purity > 99.75%), and MET (IS) (purity > 99.7%) were obtained from Centaur Pharmaceuticals Pvt. Ltd. (Mumbai, India). LCA (purity > 99.80%) and irbesartan (IS) (purity > 99.90%) were obtained from Aristo Pharmaceuticals Pvt. Ltd. (Mandideep, India). HPLC grade methanol, formic acid, ammonium formate, and orthophosphoric acid (85%) were purchased from Merck Ltd. (Mumbai, India). Ultra-pure water (18.2 M *Ω* cm) was obtained from a Milli-Q water purification system (Millipore, Milford, MA, USA). The HPLC mobile phase and sample aliquots were filtered through 0.22 μm Nylon-66 filters (Agilent Technologies, USA) before use. Oasis^®^ HLB 30 mg/1 cc solid phase extraction cartridges were obtained from Waters (Milford, Massachusetts, USA). Blank rat plasma with tripotassium salts of ethylenediaminetetraacetic acid (K3EDTA) as an anticoagulant was obtained from healthy male Wistar rats (Experimental Animal House, Department of Pharmaceutical Sciences and Technology, Birla Institute of Technology, Mesra, Ranchi).

### Instrumentation

LC-MS/MS equipment consisted of an Agilent Technologies 1200 Series liquid chromatography system equipped with a degasser (G1322A), an SL binary pump (G1312B), a high-performance autosampler (G1357D, HiP-ALSSL+), and a thermostated column compartment (G1316B SL) which was coupled with a 6460 triple-quadrupole mass spectrometer (Agilent Technologies, USA) and was operated with an Agilent G1948B ionization source in switching the electrospray ionization (ESI) mode during chromatography from positive to negative. An Agilent Mass Hunter workstation was used for the control of the equipment, data acquisition, and analysis.

### Chromatography and Mass Spectrometry Conditions

Analytes and IS were separated on an Agilent Poroshell 120, EC-C18 (50 mm × 4.6, 2.7 μm particle size) column at ambient temperature and under isocratic mobile phase conditions consisting of methanol/water (85:15, v/v) containing 5 mmol/L ammonium formate and 0.1% formic acid at a flow rate of 0.4 mL/min. The total run time was 2 min and the injection volume was 5 μL.

The MS recordings were performed by ESI with multiple reaction monitoring (MRM) to acquire the mass spectra of the compounds. The source-related parameters such as drying gas temperature (N_2_), 300°C; gas flow, 8 L/min (N_2_); nebulizer pressure, 45 psi (N_2_) were used to optimized the method. The capillary voltage was 4000 V for positive ionization and −3500 V for negative ionization. The optimized values of the analyte-related parameters are summarized in Table 1.

**Tab. 1:**
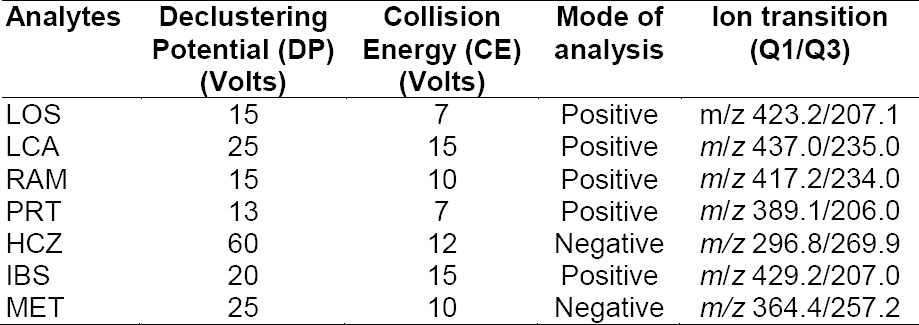
Analyte-related mass spectrometer parameters

### Preparation of Standards, Calibration Standards, and Quality Control Samples

The stock solutions of LOS, LCA, RAM, RPT, and HCZ that were used to make the calibration standards and quality control (QC) samples were prepared by dissolving an appropriate amount corresponding to 1.0 mg/mL in methanol. The stock solutions were then serially diluted with mobile phase to provide working standard solutions at the desired concentrations. The stock solutions of ISs (IBS and MET) were prepared by dissolving an appropriate amount corresponding to 1.0 mg/mL in methanol and 100 ng/mL working solutions of ISs were prepared by diluting its stock solution with mobile phase. The calibration standards for these analytes were prepared daily by spiking 10 μL of the appropriate standard working solutions to 100 μL blank Wistar rat plasma to provide the final concentrations of 3–3000 ng/mL for LOS and LCA, 0.1–200 ng/mL for RAM and RPT, and 1–1500 ng/mL for HCZ. Quality control (QC) samples at LLOQ, low (LQC), medium (MQC), and high (HQC) levels were prepared at the concentrations of 3, 9, 600, and 2700.00 ng/mL for LOS and LCA, 0.10, 0.30, 15, and 180 ng/mL for RAM and RPT, and 1, 3, 90, and 1200 ng/mL for HCZ. ISs stock solutions were made at an initial concentration of 1.00 mg/mL in methanol. The ISs working solution (100 ng/mL) was made from the stock solution using mobile phase for dilution. All solutions described above were stored at 4–8°C.

### Sample Preparation

A 100 μL aliquot of a rat plasma sample was mixed with 10 μL of internal standard (ISs) working solution (100 μg/mL of ISB and MET) and 50 μL 5% orthophosphoric acid (pH 3.8) in a 2-mL polypropylene tube. The sample was briefly vortex-mixed and then centrifuged at 5,000 rpm for 2 minutes. The pre-treated samples were loaded onto conditioned cartridges (Oasis, HLB 30 mg/1cc, Waters Corporation, USA) that were preconditioned with methanol followed by water and centrifuged (Eppendorf Refrigerated Centrifuge, 5810R) at 1500 rpm for 2 min. Plasma was drained out under nitrogen pressure and the extraction cartridge was washed with 1 mL water, 250 μL 1% orthophosphoric acid, followed by 250 μL 5% methanol. The sample was eluted by passing 400 μL of the methanol: acetonitrile mixture (50:50). The eluent was evaporated to dryness at 50°C. The dried residue was reconstituted with 250 μl of the mobile phase and transferred into an autosampler vial for injection. A 5 μL aliquot of the eluent was injected into the LC-MS/MS system for analysis.

### Method Validation

The developed method was validated for selectivity, linearity, accuracy, and intra-day and inter-day precision, recovery, matrix effects, and stability according to the Food and Drug Administration (FDA) guidelines for bioanalytical method validation [51].

#### Selectivity

Selectivity is the ability of an analytical method to differentiate and quantify the analyte in the presence of other components in the sample. The selectivity was evaluated by analyzing blank plasma samples of six different rats, blank plasma spiked with LOS, LCA, RAM, RPT, HCZ, and IS, and a rat plasma sample (pharmacokinetic samples).

#### Calibration Curve and Lower Limit of Quantification

The calibration curves of the analytes were constructed using standard plasma samples at eight non-zero concentrations. The curves were best-fitted using a least squares linear regression model y = mx + c weighted by 1/x, in which y is the peak area ratio of the analyte to IS, m is the slope of the calibration curve, c is the y-axis intercept of the calibration curve, and × is the analyte concentration. The lower limit of quantification (LLOQ) was defined as the lowest concentration point on the calibration curve with an acceptable accuracy within ±20% and the precision < 20%, using the analysis of six replicates.

#### Precision and Accuracy

Inter-batch precision and accuracy of the assay were evaluated by running three validation batches on three separate days, whereas intra-batch precision and accuracy were evaluated within a batch. Each batch consisted of one set of calibration standards and six replicates of quality control samples at three QC levels (LQC, MQC, and HQC). Precision was expressed as the percentage of the coefficient of variation (% CV = standard deviation of the measured value/mean measured value × 100), and should not be greater than 15% at each concentration level of the nominal concentration. Accuracy of the method was determined by calculating the percentage deviation observed in the analysis of QCs and expressed as the relative error (RE = measured value/true value −1), and should be within 85–115%.

#### Extraction Recovery, Matrix Effects, and Stability

The extraction recoveries of the analytes were determined by analyzing six replicates (n = 6) of rat plasma samples at LLOQ, LQC, MQC, and HQC levels and comparing the peak area of each analyte in the spiked rat plasma samples with those of samples to which the analyte had been added after extraction. The matrix effects are generally due to the influence of coeluting compounds on the actual analyte ionization process. The matrix effect was evaluated by comparing the mean peak area of the analytes spiked in blank extracted plasma samples (A) with the corresponding mean peak areas obtained by direct injection of the standard solutions of analytes (B) [matrix effect = (A/B) × 100]. For a method to be free from the relative matrix effect, the % coefficient of variation (CV) of the normalized matrix effect should be less than 15% [52, 53]. Extraction recovery and matrix effects for the ISs were also investigated at the concentration of 100 ng/mL. Drug stability in a biological fluid is a function of the storage conditions, the chemical properties of the drug, the matrix, and the container system. The stability of analytes and ISs in rat plasma was evaluated under possible conditions that should reflect situations likely to be encountered during actual sample handling and analysis, including long-term (frozen at the intended storage temperature and conditions) and short-term (benchtop and room temperature conditions) storage, autosampler stability, and after going through freeze-thaw cycles. The stability of analytes and ISs in stock solution was also evaluated.

#### Application to Pharmacokinetic Study

Pharmacokinetic studies were carried out using six young and healthy male Wistar rats weighing 240–270 g. The animals were housed in polyacrylic cages and maintained under standard laboratory conditions (temperature 25 ± 2°C) with dark and light cycles for at least seven days prior to the experiment and were given a commercial rat chow and water *ad libitum*. The experimental protocol was approved (BIT/PH/IAEC/04/2013) by the Institutional Animal Ethical Committee (621/02/Ac/CPCSEA) prior to the conduct of the animal experiments.

After an overnight fast, the rats were given an oral administration of a mixture of LOS (10 mg/kg), RAM (1 mg/kg), and HCZ (2.5 mg/kg) dissolved in 0.1% carboxymethyl cellulose. The animals had free access to water after 4 h of the oral administration of drugs. The blood (~250 μL) was collected into heparinized tubes from the suborbital veniplex before administration and at 0.08, 0.15, 0.5, 1.0, 1.5, 2.0, 3.0, 4.0, 6.0, 8.0, 12.0, 24.0, 36.0, 48.0, and 72.0 hrs after dosing. The plasma was immediately separated by centrifugation and stored frozen at −80°C until analysis.

## Results and Discussion

### Optimization of the Method

The main objective of our research work was to develop and validate a simple, sensitive, and high-throughput LC-MS/MS method having a simple extraction procedure with high recoveries and short analysis time, while retaining good efficiency from small volumes of plasma.

#### Mass Spectrometry

**Fig. 1:**
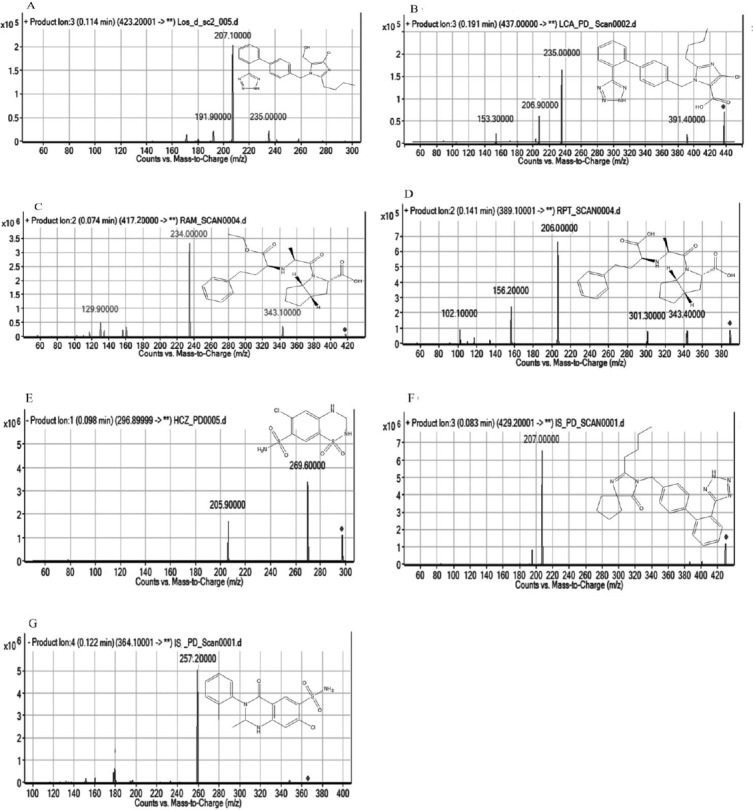
Chemical structures and product ion mass spectra of [Losartan (LOS), (A)], [Losartan carboxylic acid (LCA), (B)], [Ramipril (RAM), (C)], [Ramiprilat (RPT), (D)], [Hydrochlorothiazide (HCZ), (E)], [Irbesartan (ISB, IS), (F)], and [Metolazone (MET, IS), (G)

The positive mode of ionization was selected for LOS, LCA, RAM, RPT, and IS (IBS) because the relative abundance of precursor ions and product ions for these analytes were more in positive mode. On the contrary, the negative mode of ionization was selected for HCZ and IS (MET) because the relative abundance of precursor ions and product ions for these analytes were more in negative mode. The chemical structures and product ion mass spectra of LOS, LCA, RAM, RPT, HCZ, and ISs (IBS and MET) are shown in Fig. 1.

#### Liquid Chromatography

The feasibility of various mixture(s) of solvents such as acetonitrile and methanol using different buffers such as ammonium acetate, ammonium formate, trifluoroacetic acid, and formic acid along with altered flow rates (in the range of 0.4-0.8 mL/min) was tested for the complete chromatographic resolution of analytes and IS (data not shown). The effect of the column on the analysis of analytes and IS was also evaluated. Various columns (C8, C18, phenyl, etc.) were tried to get better results in terms of peak shape, retention time, and resolution. Finally, analytes and ISs were eluted on an Agilent Poroshell 120, EC-C18 (50 mm × 4.6 mm, i.d., 2.7 μm) column using an isocratic mobile phase consisting of methanol/water (85:15, v/v) containing 5 mmol/L ammonium formate and 0.1% formic acid at a flow rate of 0.4 mL/min. The total run time was 2 min and the injection volume was 5 μL.

#### Plasma Extraction

As LOS, LCA, RAM, RPT, HCZ, and ISs (IBS and MET) have significant differences in drug–plasma binding and physicochemical properties (pKa, solubility, stability at various pH, etc.), it was difficult to optimize the extraction procedure for these analytes from rat plasma. Several methods were tried based on the previously reported methods such as protein precipitation (PPT), liquid–liquid extraction (LLE), or solid phase extraction (SPE) for the extraction of these analytes from plasma. Finally, the SPE procedure was chosen to extract the analytes and ISs from plasma because of its high extraction efficiency, good reproducibility, and lower interference of endogenous matrix components compared to PPT and LLE.

### Method Validation

#### Selectivity

**Fig. 2:**
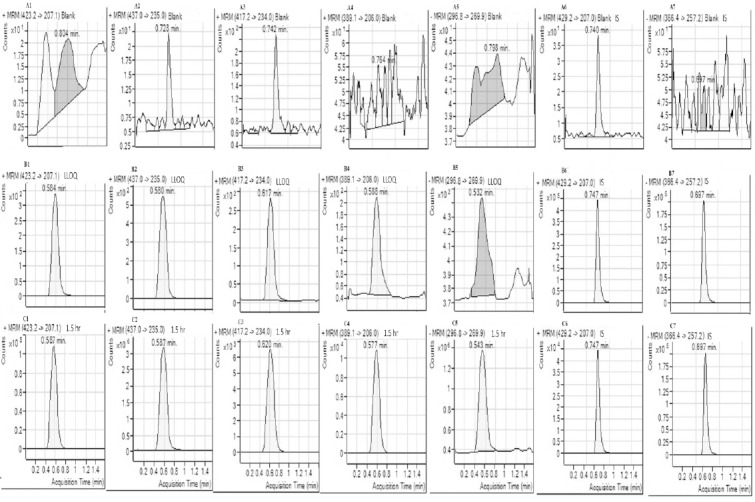
Representative MRM chromatograms of blank rat plasma samples: LOS (A1), LCA (A2), RAM (A3), RPT (A4), HCZ (A5), IS (IBS, A6), and IS (MET, A7). Representative MRM chromatograms of a blank rat plasma sample spiked with: LOS at the LLOQ of 3 ng/mL (B1), LCA at the LLOQ of 3 ng/mL (B2), RAM at the LLOQ of 0.1 ng/mL (B3), RPT at the LLOQ of 0.1 ng/mL (B4), HCZ at the LLOQ of 1 ng/mL (B5), IS (IBS) at 100 ng/mL (B6), and IS at 100 ng/mL (B7). Representative MRM chromatograms of a plasma sample of LOS (C1), LCA (C2), RAM (C3), RPT (C4), and HCZ (C5), obtained from a rat at 1.5 hr after oral administration of a mixture of LOS (10 mg/kg), RAM (1 mg/kg), HCZ (2.5 mg/kg), IS (ISB) at 100 ng/mL (C6), and IS (MET) at 100 ng/mL

The developed LC-MS/MS method demonstrated good selectivity. There were no interferences at the retention times for LOS (0.584 min), LCA (0.580 min), RAM (0.617 min), RPT (0.588 min), HCZ (0.532 min), and ISs (IBS, 0.747 and MET, 0.697 min) as shown in Fig. 2. These results supported the high specificity and selectivity of this method.

#### Extraction Recovery and Matrix Effects

The extraction recoveries (n = 6) of LOS, LCA, RAM, RPT, and HCZ from rat plasma at all QC (LLOQ, LQC, MQC, and HQC) levels were found to be more than 70.0%. The mean extraction recoveries (n = 6) of both ISs (ISB and MET) were > 88.20 ± 6.20%. The matrix effects ranged from 88.99 ± 6.20 to 99.64 ± 3.14 at all QC levels of samples. The matrix effects of ISs at 100 ng/mL were 99.32 ± 2.89 for ISB and 98.45 ± 3.80 for MET. The matrix effect results showed that there was negligible ionization suppression or enhancement from the plasma matrix for this method. Results of the extraction recovery and matrix effects are shown in Supporting Information (Table S1).

**Table S1:**
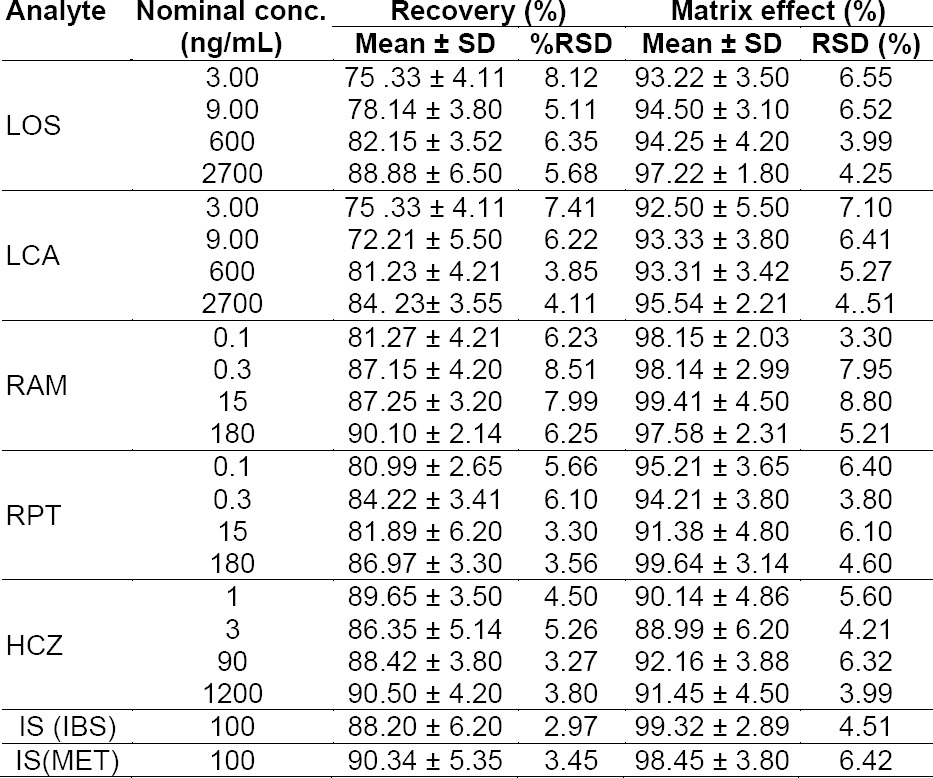
Results of recovery and matrix effect of LOS, LCA, RAM, RPT, HCZ, and ISs in rat plasma (*n* = 6)

#### Sensitivity and Linearity

The lower limit of quantitation (LLOQ) of the assay was 3 ng/mL for LOS and LCA, 0.1 ng/mL for RAM and RPT, and 1 ng/mL for HCZ with acceptable accuracy and precision. The plasma calibration curve was constructed using eight calibration standards (viz., 3–3000 ng/mL for LOS and LCA, 0.1–200 ng/mL for RAM and RPT, and 1–1500 ng/mL for HCZ). The calibration curve was prepared by determining the best fit of the peak-area ratios (peak area analyte/peak area IS) vs. concentration, and fitted to the y = mx + c using a weighing factor (1/x). The representative regression equation for the calibration curve was y = 0.6348x + 0.0297 for LOS; y = 0.0791x – 0.0345 for LCA; y = 6.6271x – 4.1289 for RAM; y = 0.1463x + 0.0027 for RPT; and y = 0.0022x + 9.4470 for HCZ, with a correlation coefficient of > 0.9995 for all analytes.

#### Precision and Accuracy

The precision and accuracy of the developed method were determined by analysis at three levels of quality control (LQC, MQC, and HQC) samples. Intra-batch and inter-batch precision were found to be less than 10% for all analytes. Accuracy was expressed as the percent relative error (%RE) for all analytes. The intra- and inter-run precision (RSD, %) and accuracy (RE, %) results for all analytes are shown in Table 2.

**Tab. 2:**
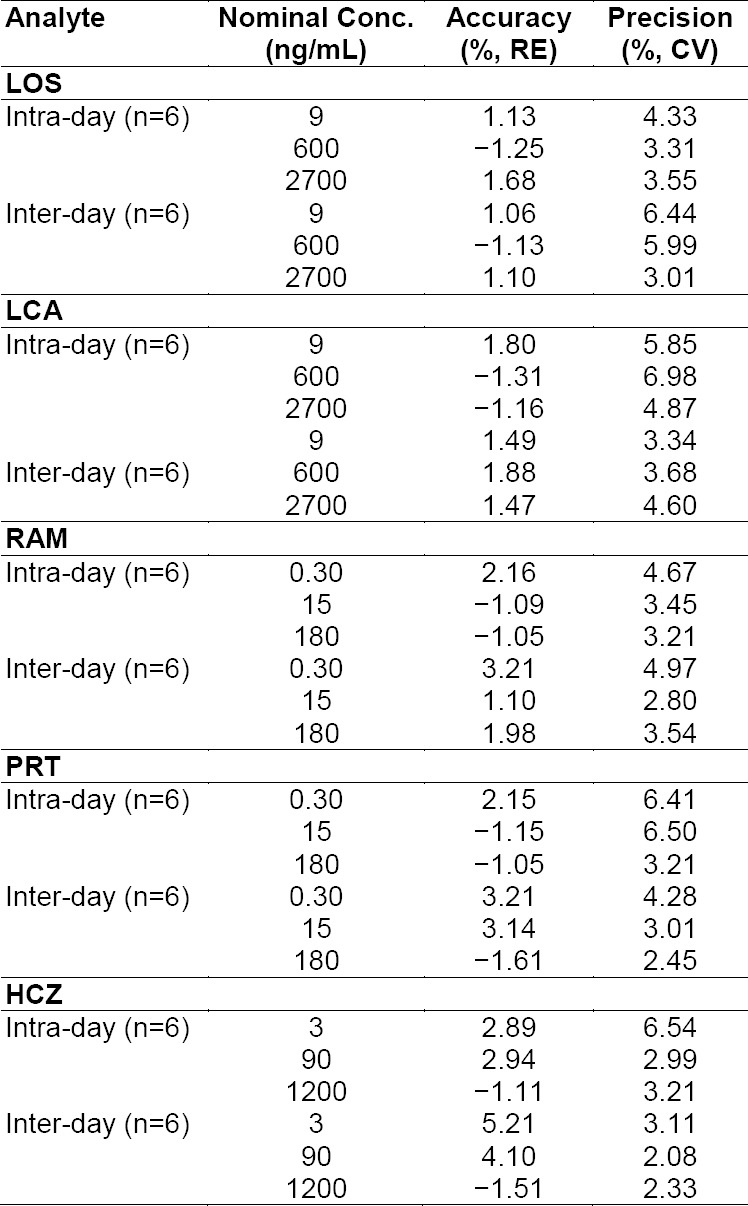
Intra- and inter-day precision and accuracy in determination of LOS, LCA, RAM, RPT, and HCZ in rat plasma

#### Stability Studies

The stability studies of all analytes and ISs were performed at three QC concentration (LQC, MQC, and LQC) levels in six replicates (n=6). The stability in rat plasma was evaluated under different temperature and storage conditions, such as: short-term stability at room temperature for 12 h (benchtop stability), long-term stability at −80°C for 90 days, three freeze-(−80°C)-thaw-(room temperature)-cycles on consecutive days. Post-preparative stability was evaluated by keeping extracted QC samples in the autosampler at 4°C for 24 h. The stability of stock solutions (4−8°C for 7 days) of all analytes and ISs was also evaluated. The results of stability studies are shown in Supporting Information (Table S2).

**Table S2:**
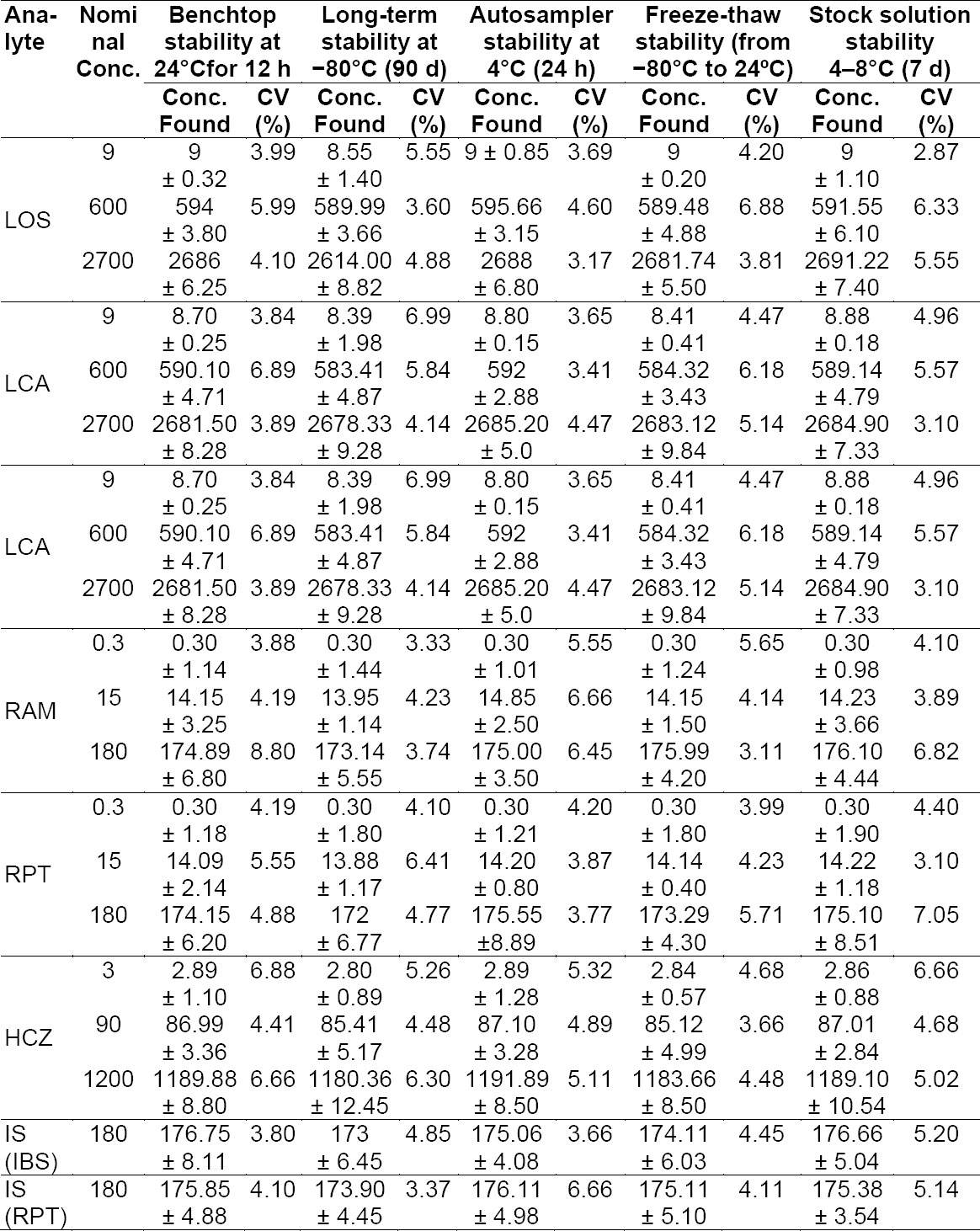
Stability of LOS, LCA, RAM, RPT, HCZ, and ISs after storage under indicated conditions (n=3); Concentrations in ng/mL (± SD).

### Application to Pharmacokinetic Study

**Fig. 3:**
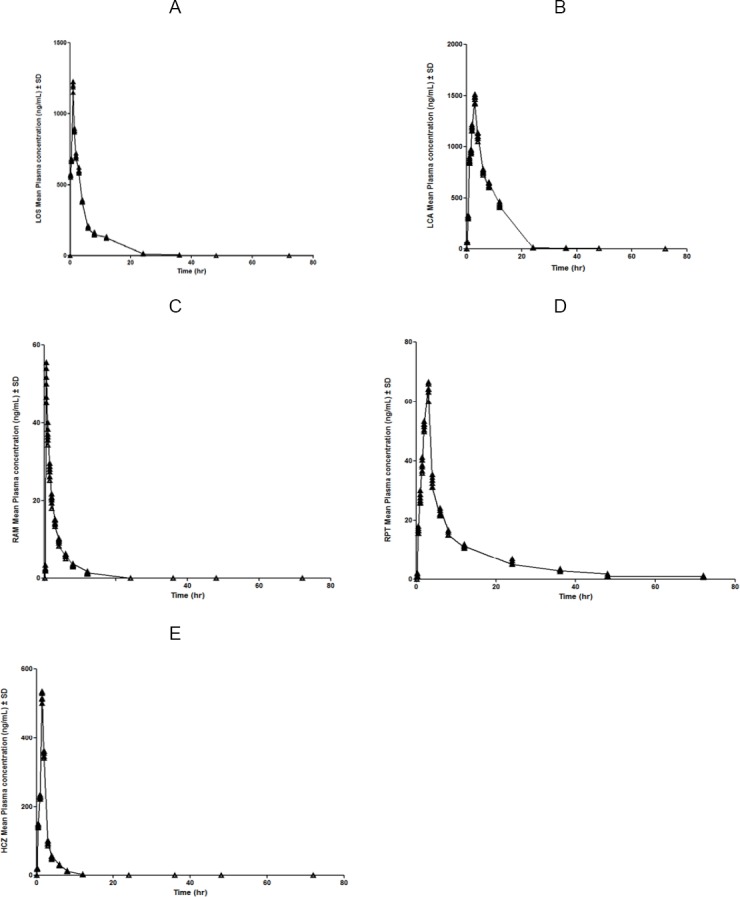
Mean plasma concentration vs. time curve of LOS (A), LCA (B), RAM (C), RPT (D), and HCZ (E) after oral administration of a mixture of LOS (10 mg/kg), RAM (1 mg/kg), and HCZ (2.5 mg/kg) in rats

The method described above was successfully applied to study the pharmacokinetics of MET, RAM, and RPT in six male Wistar rats after an oral administration of the mixture of LOS (10 mg/kg), RAM (1 mg/kg), and HCZ (2.5 mg/kg). Pharmacokinetic (PK) analysis was performed using the software Phoenix WinNonlin^®^ software (version 6.3; Certara USA, Inc., St. Louis, USA) on the rat plasma data of MET, RAM, and RPT, using non-compartmental methods modified for sparse sampling which provides additional information of standard error for the area under the curve, AUC_last_ [54]. The concentrations below quantifiable limits were converted to zeroes prior to pharmacokinetic analysis [55]. A representative chromatogram of a post–dose sample after 1.5 hrs is shown in Fig. 2. The resulting mean plasma concentration–time curves of LOS, LCA, RAM, RPT, and HCZ is shown in Fig. 3. The main pharmacokinetic parameters are summarized in Table 3.

**Tab. 3:**
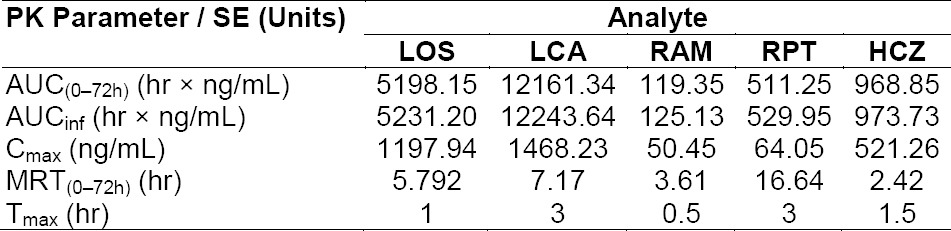
Pharmacokinetic parameters of LOS, LCA, RAM, RPT, and HCZ obtained after an oral administration of a mixture of LOS (10 mg/kg), RAM (1 mg/kg), and HCZ (2.5 mg/kg)

## Conclusion

In this paper, a novel LC–MS/MS method for the simultaneous determination of LOS, LCA, RAM, RPT, and HCZ in rat plasma is described. This method was successfully applied to the pharmacokinetic study after an oral administration of a mixture of LOS (10 mg/kg), RAM (1 mg/kg), and HCZ (2.5 mg/kg) in rats. The developed method has several advantages as compared to the methods reported in literature such as simple sample preparation procedures by SPE, short analysis time (2 min per sample), and high sensitivity which rendered the method fitting for the purpose of its application to measure concentration-time profiles for bioavailability, pharmacokinetic, bioequivalence, and drug–drug interaction studies of LOS, LCA, RAM, RPT, and HCZ for routine therapeutic drug monitoring.

## Supporting Information

Data of the extraction recovery and matrix effects (Table S1) as well as the results of the stability studies (Table S2) are available in the online version (Type: PDF, Seize: < 0.1 MB): http://dx.doi.org/10.3797/scipharm.1410-15.
